# An exploratory randomised controlled trial of a premises-level intervention to reduce alcohol-related harm including violence in the United Kingdom

**DOI:** 10.1186/1471-2458-12-412

**Published:** 2012-06-07

**Authors:** Simon C Moore, Simon Murphy, Susan N Moore, Iain Brennan, Ellie Byrne, Jonathan Shepherd, Laurence Moore

**Affiliations:** 1 Violence & Society Research Group, School of Dentistry, Cardiff University, Cardiff CF14 4XY, UK; 2 Cardiff Institute for Society and Health, Cardiff School of Social Sciences, Cardiff University, 1-3 Museum Place, Cardiff CF10 3BD, UK; 3 Department of Social Sciences, University of Hull, Cottingham Rd., Hull, HU6 7RX, UK

**Keywords:** Violence, Alcohol, Licensed premises, Exploratory trial, Intervention

## Abstract

**Background:**

To assess the feasibility of a randomised controlled trial of a licensed premises intervention to reduce severe intoxication and disorder; to establish effect sizes and identify appropriate approaches to the development and maintenance of a rigorous research design and intervention implementation.

**Methods:**

An exploratory two-armed parallel randomised controlled trial with a nested process evaluation. An audit of risk factors and a tailored action plan for high risk premises, with three month follow up audit and feedback. Thirty-two premises that had experienced at least one assault in the year prior to the intervention were recruited, match paired and randomly allocated to control or intervention group. Police violence data and data from a street survey of study premises’ customers, including measures of breath alcohol concentration and surveyor rated customer intoxication, were used to assess effect sizes for a future definitive trial. A nested process evaluation explored implementation barriers and the fidelity of the intervention with key stakeholders and senior staff in intervention premises using semi-structured interviews.

**Results:**

The process evaluation indicated implementation barriers and low fidelity, with a reluctance to implement the intervention and to submit to a formal risk audit. Power calculations suggest the intervention effect on violence and subjective intoxication would be raised to significance with a study size of 517 premises.

**Conclusions:**

It is methodologically feasible to conduct randomised controlled trials where licensed premises are the unit of allocation. However, lack of enthusiasm in senior premises staff indicates the need for intervention enforcement, rather than voluntary agreements, and on-going strategies to promote sustainability.

**Trial registration:**

UKCRN 7090; ISRCTN: 80875696

## Background

The health costs of alcohol misuse in the United Kingdom (UK) are rapidly increasing and estimated to be between 2% and 5% of gross domestic product [1,2]. Between 1997 and 2005/6, admissions to the UK’s National Health Service (NHS) for mental and behavioural disorders due to alcohol, alcohol-related liver disease and toxic effects of alcohol doubled [3]. It is estimated that alcohol related disease accounts for one in eight NHS bed days [3] and between midnight and 5 am approximately 70% of A&E admissions are alcohol-related [3].

Premises licensed to sell alcohol are prime locations for alcohol-related harm, including violence, suggesting that premises-level [4-6] interventions designed to reduce risk [7] provide one potentially cost-effective [8] and feasible policy approach [9]. The 2003 UK Licensing Act affords police and other practitioner’s greater legal and regulatory powers to intervene in problematic premises and motivates a harm minimisation approach in Designated Premises Supervisors (DPS), the named senior staff member responsible for a licensed premises. Accordingly, a number of promising premises-level interventions to promote a more proactive approach to harm reduction have been identified [10].

Evidence of effectiveness is limited, with very few evaluations employing a randomised controlled trial (RCT) design; none have been conducted in the UK. Further, outcome measures are mostly observational [7] or secondary to alcohol misuse (e.g. drunk driving and alcohol-related car accidents rather than objective measures of alcohol consumption). When objective measures of alcohol misuse have been used, such as breath alcohol concentration (BrAC) [11,12], studies have typically focused on specific sub-populations, such as students [13], and not on the general drinking population. Disparate measures of violence have been used, including data from hospital Emergency Departments (ED) [14] and the local police [15], yet their validity has not been assessed at the premises-level [16]. Concerns have also been raised regarding barriers to intervention implementation and levels of acceptability of a risk management approach in licensed premises. There is a need to determine effective and feasible intervention strategies in the UK [17]. There is a particular need to improve what is known about the theoretical basis for such interventions, to define trial methods and protocols [18,19], define and test recruitment strategies, determine appropriate outcome measures [20], assess effect sizes, and test intervention feasibility. Such an approach reflects standard developmental frameworks for the evaluation of complex interventions and reinforces the need for feasibility studies such as the one reported here [21].

Interventions in licensed premises have taken a number of formats across studies and include server training and responsible beverage service, increasing police presence in the night time economy (NTE) and risk-led interventions (RLI) [22]. Interventions that focus on a single risk, such as responsible beverage service, fail to account for the complex relationships between staff (i.e. servers, security and management) and the premises environment that can together affect harm reduction. While the effectiveness of interventions in premises is poorly understood, studies that have evaluated RLIs suggest they are successful [23,24].

Having previously identified successful approaches to intervention targeting, study recruitment methods and the development of appropriate outcome measures and data collection techniques [11,12,20,25], this paper outlines the theoretical basis and evaluates implementation of a RLI. It also assesses intervention effect sizes, sampling bias and while cost-effectiveness was not a focus of the study, provides some provisional estimates to inform future development.

### Intervention theory and implementation

The RLI [18] developed for use here was informed by three previous projects [24,26,27]. Intervention content was developed from an extensive literature that documents those features of premises environments that contribute to harm [7,22,23]. These interventions typically involve an initial risk audit [23] followed by an action plan [23,24]. If the action plan is adopted risks that promote premises-level harm will have been addressed and a reduction in alcohol-related harm is expected. Action plans require premises to make changes to operating procedures (e.g. reducing capacity, changing how security staff are deployed, checking patrons’ age at the door), improve staff training, as well as covering aspects of the internal and the external environment, such as addressing poor visibility.

**Figure 1:**
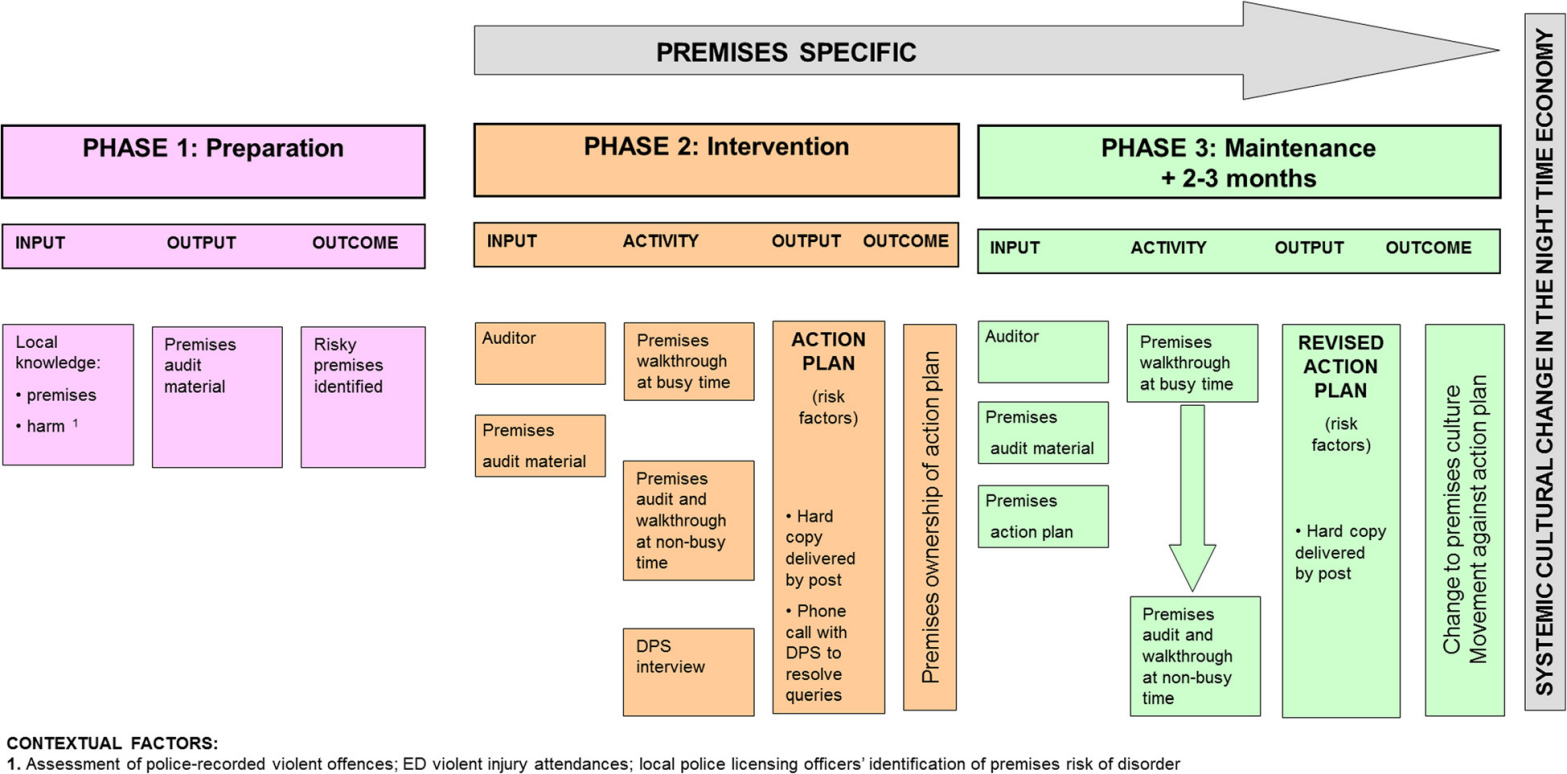
Initial intervention logic model.

Figure 1 represents the logic model for the planned intervention. A preparation phase involved refining the audit tool and was conducted in collaboration with independent professional auditors who were experienced in evaluating the policies and processes within licensed premises. The same auditors conducted the audit, although they had no statutory authority to impose change on premises. Premises-level risk factors for intoxication and disorder were assessed in two premises walkthroughs, once during the day when quiet and once during the evening with customers present, and one face to face interview with the premises manager. Major categories of risks covered the external environment immediate to the premises, staff (e.g. levels of training), customer behaviour, the internal physical environment, operational procedures and security measures. Results from the audit informed a bespoke premises action plan, delivered to premises managers in the experimental condition as a written report that identified risk factors and suggested solutions. Premises managers were telephoned one week later to ensure the action plan had been received.

Risk factors are those characteristics of a licensed premises that are associated with an increased likelihood of severe intoxication and disorder. These factors are many, varied and unequally weighted. Interactions between factors further mitigate and inflate risk in different ways. Furthermore, it is likely that many risk factors have not yet been described or that latent factors may offer a simpler explanation for clusters of the observed risk factors. The theoretical frameworks that motivated our approach were Routine Activity Theory (RAT) [28] and ‘Broken Windows’ theory (BWT) [29,30], which describe factors that are necessary or that increase the likelihood of crime taking place in certain situations. The researchers applied existing research on premises-level risks together with research evidence of the effect of alcohol on cognition and behaviour – among offenders and victims to RAT and BWT to identify potential risk factors for disorder in licensed premises. RAT [28] is a situational approach to crime that emphasises the co-occurrence of three phenomena in the etiology of crime: a motivated offender, a suitable target or victim and the absence of a capable guardian. The fact that these three phenomena need to co-occur indirectly emphasises the location of the incident. This emphasis on place and time makes it a suitable theory for developing insights into violence in licensed premises. Similarly, the recognition of a victim’s participation and the failure of guardians to prevent the incident also make RAT a suitable theory in which to ground prevention research as it admits factors other than those that are offender-specific. BWT [30] suggests that offending is informed by situational cues that indicate an absence of social order such as graffiti, litter, vacant buildings and broken or boarded windows. An absence of social order indicates a lack of capable guardians, making that environment a suitable place to commit crime. In ‘real-world’ experiments, Keizer et al. [31] have shown that individuals take cues from their environment to inform behaviour. For example, the sound of fireworks being set off illegally was related to an increase in littering, and the presence of graffiti on a mailbox was related to an increase in opportunistic theft.

The wide variety of activities available in NTEs brings with them a range of risk factors for disorder and intoxication and numerous studies have sought to identify those risk factors. These risks are summarised briefly below; for more detailed accounts see comprehensive reviews by Graham and Homel [23], and Green and Plant [7].

#### External management of customers

The entrance and façade of a premises informs potential customers of the characteristics within. Based on these characteristics customers can predict music policy, other customers’ characteristics and crowd density. While it is unclear whether customers can accurately predict risk of disorder in a premises, it is likely that the interpretation of premises’ social norms are informed by these external characteristics. Therefore, door staff behaviour, queue management and the management of intoxicated or disorderly customers are fundamental in providing cues about descriptive and injunctive norms. Not only do the external characteristics of a premises inform customers about levels of permissiveness, rule-breaking and disorder, interactions outside the premises also represent potential flashpoints for disorder. Lastly, the congregation of people outside premises after closing time is a risk factor for disorder. Often by this stage door staff will have finished their work on the door and their attention has shifted to the interior where they are encouraging customers to vacate. This leaves the external area of the premises without a designated guardian at a time when customers are likely to be most intoxicated.

#### High outlet density

The distribution of licensed premises in urban centres has been identified as a key contributor to levels of alcohol-related harm [32]. Areas with high concentrations of licensed premises have disproportionately higher levels of disorder, suggesting a cumulative, non-linear effect of outlet density [33]. Clearly there can be no strict association between outlet density and harm as areas with a large number of premises but few patrons would be expected to exhibit low levels of alcohol-related harm. High density most likely encourages behaviours that are associated with harm, such as pub-hopping, competition between premises and therefore inappropriate promotions. Therefore, while outlet density is not in itself in the control of premises staff, it would be possible for premises to mitigate those features that are causally associated with harm and are correlated with heightened outlet density, such as crowding and competition [34].

#### Appropriate and vigilant security staff

As door staff represent both the expression and actuality of guardianship in a licensed premises, it is important that they are adequately trained and present a professional and welcoming demeanour. In England and Wales it is illegal, under the Licensing Act, to allow disorderly conduct on premises. Furthermore, any member of staff who is authorised to prevent disorder and allows it to happen on the premises is legally culpable. Therefore, refusing admission to prospective customers exhibiting risky behaviour is regarded as a main role of door staff in England and Wales. It is also illegal to sell alcohol to people under the age of 18 years and although it is the server who is ultimately responsible, scrutinising prospective customers’ age is also a role of door staff. Door staff can use a variety of tactics in refusing admission to disorderly or underage patrons. These include asking for proof of age, employing the “Think 21” and “Think 25” schemes, each scheme requires that door staff request ID if the patron looks to be under the age of 21 or 25 years of age respectively, and observing customers for signs of intoxication as they approach the premises. As the primary guardians and most visible place managers [35] for a licensed premises, it is essential that a sufficient number of trained door staff are employed. Further, their deployment should be sensitive to the number of premises entrances, the number of expected customers and past history of disorder. It is a legal requirement, under the UK 2001 Private Security Industry Act, that all door staff are licensed, so making them one of the few premises personnel in the UK with any formal professional requirement governing their role. To ensure that this legal requirement is met, many premises maintain a door staff register requiring door staff sign in and sign out for work shifts, provide their unique license code and its expiry date. In order to obtain this license, applicants are required to complete an examined training course. Moreover, in the event of a violent incident, customer ejection or injury on the premises, the details of the incident should be recorded clearly, accurately and promptly in a log book and, in the UK context, reported to Environmental Health Officers. This allows premises managers to explore trends in disorder and to determine how to best allocate door staff.

#### Appropriate and vigilant serving staff

Premises serving staff play a role in the safe service of alcohol and the prevention of disorder as they are responsible for refusing service to underage customers, intoxicated customers and identifying signs of disorder within the premises. It is essential that serving staff are aware of their legal responsibilities and that they take these responsibilities seriously. Server training has been shown to have limited, short-term effects in improving serving practices in respect of the refusal of service to intoxicated patrons [22]. As serving staff act as unofficial place managers, it is important that sufficient numbers of staff are deployed to facilitate these serving practices. Insufficient numbers of serving staff is associated with increased levels of disorder in a premises as it increases competition for service between customers [5] and crowding [34]. Furthermore, a premises with a high proportion of male staff is associated with disorder in licensed premises [36], although this phenomenon may be a reaction to past disorder as opposed to a causal factor. Clarke [37] further suggests that an overly sexualised dress code for female serving staff can contribute to heightened levels of arousal in a premises and this is further implicated in disorder.

#### Environmental factors

A number of studies have sought to identify environmental factors that influence the likelihood of disorder. Graham et al. [38] conducted a detailed multilevel analysis of risk factors for bars in Toronto, while Green and Plant [7] have collated a detailed description of these risk factors. Evidence suggests that showing sport in premises increases the length of customers’ visits [39] and is associated with an immediate increase in levels of aggression. Music is related to levels of disorder and intoxication. For example, poor quality bands can be an irritant [40] while slow-tempo music can be associated with increased drinking speeds [41]. In addition to acting as an irritant, loud music may further impair communication between intoxicated patrons, hindering the de-escalation of fractious encounters; and hinder communication between premises staff, impeding their ability to act proactively. A range of other environmental factors can act as irritants such as poor air quality [5,42], increased temperature [43-45], crowding [40], and uncomfortable furniture [5,46]. Moreover, low lighting levels reduce the capacity for formal and informal surveillance by place managers and it impairs communication and increases the likelihood of collisions between customers in the premises. Similarly, occluded areas that are not routinely monitored by guardians increase opportunities for patrons to engage in illicit and disorderly behaviour.

Glasses and bottles are frequently the most accessible weapons in premises and coupled with the level of harm that they can cause, make glassware in bars a significant risk factor for serious injury [47]. Furthermore, the presence of empty glasses and other litter on tables may signal low levels of social order, increasing the risk of violence; numerous studies have reported an association between untidy premises and disorder [26,36,40,46,48].

#### Promotions

Stockwell, Lang and Rydon [49] found that alcohol promotions were associated with intoxication but not associated with the risk of alcohol-related harm. More recently, however, studies suggest that promotions and becoming drunk are associated with police-recorded violence [20].

#### Customer behaviour and characteristics

Disorderly customer behaviour, according to BWT, contributes to perceptions of a permissive environment, increasing the likelihood of further disorder. However, the relationship between patron gender ratio and risk of disorder is unclear. Males are at far greater risk of violence than females, suggesting that violence is more likely in premises with greater proportions of males. However, evidence to support this is scarce [23]. An imbalance in the ratio of men to women in a drinking environment might be a risk factor with men competing for a scarce resource. Whether women aggressively compete with one another for male attention is not clear. While a younger age is associated with increased risk of violence, the age of premises customers is not a strong predictor of disorder on a particular night. It is likely that age interacts with several other factors, such as premises type, to contribute to disorder. Typically, persistent offenders who are frequently violent when intoxicated [50] become well known, emphasising the need for door staff and premises managers to share data across premises in an area.

## Methods

**Figure 2:**
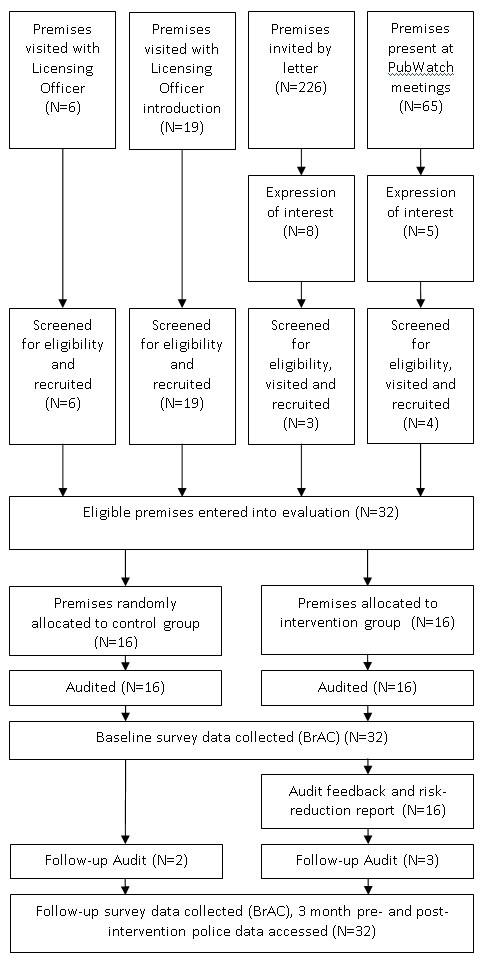
Flow diagram.

The study was based in five UK towns, one large cosmopolitan city that attracts drinkers from across the UK, a large city with a traditional NTE, and three smaller towns with well-defined NTE areas (two towns are in close vicinity and treated as one area in later analyses). Participation was voluntary and premises had the opportunity to withdraw from the study at any point. Thirty-two target premises were recruited [18] that represented those premises that had the highest levels of disorder in each of the four recruitment areas and comprised standard public houses, high volume vertical drinking establishments (typically a large bar with limited seating and no dance floor) and night clubs. The design was an exploratory two-armed parallel cluster randomised trial with embedded process evaluation in which premises were the unit of allocation. All aspects of the project methods, the intervention and analytic strategy, including the process evaluation, were published prior to study completion [18] and the study was reviewed and approved by the Medical and Dental Research Ethics Committee, Cardiff University (MDSREC 08/11). Figure 2 presents the study flow diagram; Additional file 1: Appendix 1 provides the street survey used to collect some of the data referred to here.

### Exploratory trial

#### Inclusion/exclusion criteria

Eligible premises had been associated with at least one police-recorded or ED-recorded violent incident in the twelve months up to premises recruitment (one premises was regarded as high risk by local practitioners and was therefore included even though it had no recorded incidents). The 32 premises were allocated to matched pairs. Matching was achieved through use of four pieces of information available at recruitment: the number of recorded incidents in the twelve months leading up to the start of the project (P); two exposure variables, hours open past 11 pm (T) and capacity (C); and premises location. We combined P, T and C to provide an index of risk for each premises, under the assumption that T and C are externalities inversely related to P, using R_i_ = P_i_/(T_i_C_i_), where R is the measure of risk (mean R = 0.54, SD = 1.09). Premises were matched to their nearest neighbour using R by location [18].

#### Randomisation

Each of a matched pair of premises was remotely randomly allocated to either the intervention or control condition by two independent researchers (Researcher A and Researcher B). Each premises file was placed in a sealed envelope and for each matched pair assigned the number “1” or “2” by Researcher A. The sealed envelopes were then given to Researcher B and Researcher A generated a random number independently. The random number was relayed by telephone to Researcher B. For each matched pair of premises, if an odd number was generated then the premises identity in the envelope marked 1 was allocated to the control group. If an even number was generated, the premises identity marked 2 was allocated to the intervention group. Each premises had a 50% chance of being in either control or intervention group. The research team members who were responsible for the analysis of effects sizes were blind to the intervention condition, as were all data collection staff. Since part of the process evaluation required asking different questions of intervention and control premises representatives, the research team members who conducted the process evaluation were necessarily unblinded. As matching was incorporated into the study to reduce possible imbalances between treatment arms expected with a small sample size, it was not accounted for in subsequent analyses due to the loss of degrees of freedom [51].

#### Control treatment

The premises in the control group received treatment as usual [52]. In this context, premises were subject to normal police and local authority practices in the management of licensed premises.

#### Participants

**Premises** Sixteen pairs of premises provide 80% power to detect a 0.8 standard deviation difference in premises-level rates of intoxication and violence using a two-tailed alpha of 0.05. The study was an exploratory trial and a significant effect was not anticipated.

##### Premises customers

In previous studies, where questionnaires have been considerably longer than the one used here (Additional file 1: Appendix 1), two pairs of surveyors surveyed on average 40 drinkers over five hours on Friday and Saturday evening [53]. These data are sufficient (n ≈ 120) to assess outcome measures and provide an indication of premises serving practices and, across all premises, provide sufficient data to evaluate outcome measures, methods and any future definitive trial.

#### Primary outcome measures

The primary outcome measures used in the study (police data and alcometer scores of premises’ customers) were selected for their appropriateness and feasibility in identifying and accurately associating violence and severe intoxication with study premises. Surveyor rated intoxication was used as a secondary measure and to test for sampling biases (see below). Respondent self-report violence and a measure identifying hazardous alcohol use were used to test for changes in the underlying population frequenting premises pre- and post-intervention. Identical outcome measures were used at baseline and at the three month follow up street surveys (see Additional file 1: Appendix 1).

##### Violence

Anonymised data on recorded assaults for the period 1st March 2008 to 28^th^ February 2009 were extracted from a police data base (NISHE RMS). Police data represent the most cost-effective available data on violence against the person in an area. Through exploration of these data it was possible to identify the location of recorded incidents at the premises-level. Any violence against the person that was associated with a study premises (i.e. inside or immediately outside the premises) was counted as an incident.

##### Breath alcohol concentration

BrAC was collected from respondents to the street survey using Lions Labs SD400 alcometers, calibrated to ±3 μg alcohol/100 ml breath and following manufacturer’s guidelines.

#### Secondary outcome measures

**Self report violence** Screening respondents who frequent study premises for their past experiences of violence and alcohol misuse provides a pragmatic method of assessing the dispersal due to the intervention [54-56]. The study, as designed, had no means to track premises’ customers across premises as no identifiable information was collected from respondents in the street survey. Moreover, measures of violence were necessarily premises-specific and thus any measureable effect of the intervention might be interpreted as being due to those premises customers who are prone to aggression changing their drinking venue. Previous studies of violent offenders suggests that the strongest predictor of future violence is having been violent in the past [57] and that, at least over a twelve month period, individuals’ propensity for violence is fairly stable. From this we inferred that self-report experience of violence might provide a reasonable means to characterise the proportion of those prone to violence in a given premises population. Three questions, used previously in street surveys [58], were therefore included in the street survey to capture these data (questions V1 to V3, Additional file 1: Appendix 1).

##### Fast Alcohol Screening Test

As with violence, any change in the proportion of the underlying population associated with an intervention premises and, who chronically misuse alcohol could explain any changes in outcome measures associated with the intervention. To capture this possibility the Fast Alcohol Screening Test (FAST) was included in the street survey. The FAST was originally developed for use in busy locations, such as emergency departments, to screen patients for hazardous drinking. Its brevity makes it well suited for use in a street survey of the NTE where surveyors have limited time with respondents. The FAST [55] (questions F1 to F4, Additional file 1: Appendix 1) has been previously validated in a alcometer survey of male students [56], although it has yet to be used in a survey of the NTE more generally. It is possible to validate the FAST against alcometer scores and therefore if a reduction in BrAC is observed in customers of intervention premises with FAST scores remaining stable then it would be reasonable to assume that those at risk of misuse have curtailed their consumption rather than the intervention dispersing risky drinkers.

##### Estimated intoxication

A requirement of premises staff, under the 2003 Licensing Act, is that they refuse access to premises to those who are intoxicated and to refuse service to those who become intoxicated while on premises. These judgements by premises staff will be made using subjective judgements of intoxication, not alcometer scores. We therefore included subjective estimates of intoxication (staggering gait, glazed eyes and slurred speech, and further estimated overall drunkenness along a 10-point Likert scale [12]) as secondary outcome measures of intoxication in the street survey (questions W1 to W4, Additional file 1: Appendix 1). Surveyors estimated both respondent and non- respondent levels of intoxication using four validated indices [12]. Having such estimates for those who refused to participate in the street survey allows comparisons between respondents and non-respondents and therefore possible sampling biases can be determined.

#### Procedure

**Street survey** Study premises were surveyed for five hours up until 30 minutes after they closed. The street survey methods replicated previous alcometer surveys [12]. A pair of matched premises were surveyed each night with one pair of surveyors allocated to each premises. Surveyors recruited every seventh individual walking past a designated sampling landmark near to each study premises [59], requested respondents’ past and next intended drinking locations and completed the FAST [55]. All survey responses were completed by the surveyors on behalf of respondents. On completion, respondents were asked to provide a BrAC reading using the alcometer by one surveyor. Once the respondent had left the vicinity, the other surveyor rated them on the four descriptors used to identify drunkenness: gait, eyes and speech [12] and overall drunkenness along a 10-point Likert scale.

#### Analytic strategy

Analytic strategies to assess PL violence-related harm and determine intervention effect sizes have received little attention [20,22]. The underlying assumption of PL interventions is that PL risks increase the likelihood of violence and therefore injury. However, both police and ED data are proxies to violence, recording varying aspects of the incident. One single violent incident can lead to multiple arrests and/or multiple victims and the correspondence between what is recorded and the event that produced the incident is not always clear. A premises that registers five incidents in police data is not necessarily more risky than a premises that registers one, and under conditions of moderate rarity such biases could profoundly affect inferences particularly if there is any systematic relationship between the nature of incidents and premises type. As the primary interest is premises-level risk it is therefore reasonable to assume that risks persist across a premises opening hours and therefore multiple incidents in one session can be assumed to partly reflect the presence of underlying risk. In other words, we must moderate our assumptions as we can only go as far to state that the presence of *one or more* recorded incidents in police or ED data suggests the presence of increased premises-level risk in a single session. We therefore assumed that *one or more* violent incidents indicated that for that session (defined as the period the premises was open continuously) premises-level risk was elevated. Over successive days we therefore had available data for each day and for each premises indicating whether each premises had one or more incidents on each day, or more formally whether a premises was in a state of failure (i.e. a day in which one or more incidents had occurred). Typically, studies have previously aggregated across arbitrary time periods to assess the impact of interventions [22]. However, premises-related incidents might be related to specific events, such as sporting events, and would be expected to fall to zero if premises close temporarily (e.g. for refurbishment). These events are time-specific, particularly for temporary closure which is a form of censoring, and should be made explicit in any analytic strategy. While Poisson models can accommodate aggregate count data and would normally be suitable, in order to account for potential time varying covariates, censoring, multiple events and discontinuous risk intervals, the preferred approach was to use an Andersen-Gill model [60], a derivation of the Cox proportional hazards model [20,61,62] used in the analysis of recurrent failure data. For street survey data, standard multilevel mixed-effects linear regression models (premises nested in location) were specified [63].

### Process evaluation

#### Participants and procedures

The process evaluation employed a framework described by Steckler and Linnan [64] to explore the implementation, fidelity, acceptability and sustainability of the intervention. A focus group was conducted with intervention auditors that gathered data to revise the initial theoretical logic model (Figure 1) of the intervention. Fifteen national and local stakeholders were also recruited using a theoretical sampling strategy and snowball recruitment [25,65] to access contextual factors that might influence the implementation of the intervention. In addition, face-to-face semi-structured interviews designed to assess the acceptability and implementation of the intervention were conducted with a nominated representative from intervention premises (n = 12).

#### Analytic strategy

All interviews and focus groups were digitally recorded except in two intervention premises and two stakeholders who did not wish to be recorded. In these cases, handwritten notes were taken. Digital recordings were transcribed, anonymised, and uploaded onto a software package for qualitative analysis (QSR NVivo Version 8). A grounded approach to analysis was used and researchers were blind to the RCT outcomes and were not involved in the delivery of the intervention.

## Results

### Exploratory trial

#### Effect sizes for primary and secondary outcomes

**Figure 3:**
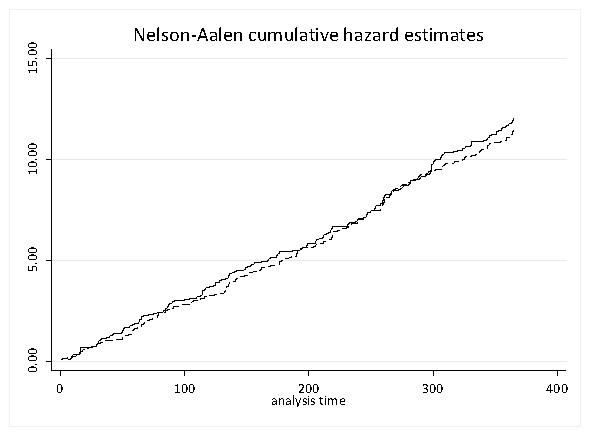
**Cumulative hazard estimates comparing two groups of sixteen premises matched according to capacity. **The vertical axis shows the Nelson-Aalen estimate, a non-parametric estimate of the cumulative hazard and the horizontal axis is time in days for twelve months of data.

**Violence** Data were available from 29 February 2008 up to delivery of the intervention and then for a three month follow-up period. The initial twelve months of data up to project start were used to assess how well premises had been matched by looking at differences in failure rate. In total, and across the 32 matched premises, 660 failures were recorded (median = 12.5). Premises were described by a binary variable such that intervention premises were allocated 1, otherwise 0. If matching was successful then the expectation is that these two groups of sixteen premises should yield no notable differences in hazard rate across this twelve month baseline period. Visual inspection of the cumulative hazard estimates (Figure 3) suggest that these two groups were indeed closely matched, accordingly the Andersen-Gill model did not provide any significant difference between them (z = 0.30). Visual inspection of Figure 3 further suggests that no observable cyclical variation in hazard rate exists for these study premises.

**Figure 4:**
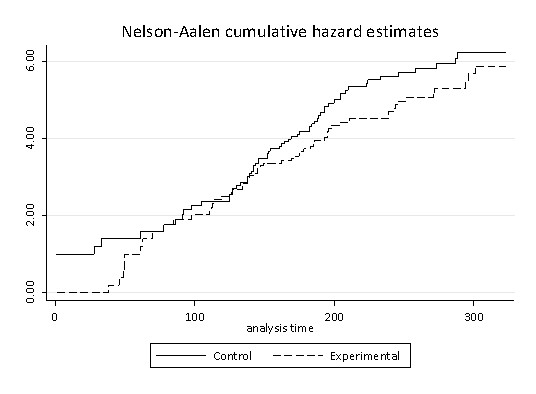
**Cumulative hazard estimates for control (dashed) and experimental (solid) premises. **The vertical axis shows the Nelson-Aalen estimate, a non-parametric estimate of the cumulative hazard and the horizontal axis is time in days.

We restricted police data to a 181 day period for each premises, 90 days pre-intervention and 90 days post intervention so that pre- and post intervention periods were symmetrical (time constraints limited the follow-up period to 90 days). The intervention was treated as a time varying covariate, coded as zero for the control group and zero up to intervention delivery in the experimental group and one thereafter. Failures were assumed to be independent and unordered. Failure rates were as follows. For the Control Group, pre-intervention = 0.0196 and post-intervention = 0.023. Whereas for the Intervention Group, pre-intervention = 0.0164 and post-intervention = 0.0147. Consulting the Nelson-Aalen cumulative hazards estimate for the control and experimental groups suggests that the two groups were broadly similar up to the midpoint, when the intervention was delivered, and thereafter a modest and sustained difference between control and intervention premises is observed post-intervention (see Figure 4).

**Figure 5:**
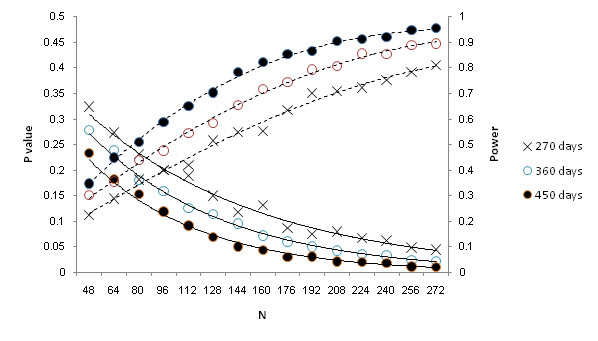
Power curves (dashed lines) and related significance (solid lines) assuming a hazard ratio of 0.9 reduction in the premises-specific violence hazard ratio.

The Andersen-Gill model suggests a non-significant hazard ratio of 0.9 in premises-level failure rate that is attributable to a reduction in violence in intervention premises. Assuming the average probability of failure for each day across the baseline period is 0.02 for both groups, and for the intervention group during follow-up this 0.9 hazard ratio is maintained, adapting routines suggested by Feiveson [66], it is possible to use simulation in order to extrapolate from the current model and design to assess the likely sample size and follow-up period required to deploy a full trial that is sufficiently powered to detect a significant reduction in premises-level violence. Simulations were constructed with the origin set at the point of intervention delivery, assuming a hazard ratio of 0.9 intervention premises and for varying follow-up periods (from 90 days to 450 days in 90 day increments) and numbers in each group (from 16 to 272 in increments of 16) with two groups, one experimental and one control group. Each datum in Figure 5 was constructed from 1,000 simulated experiments with levels of significance derived from the same Andersen-Gill model used to assess the exploratory data.

Referring to Figure 5, three follow-up periods offer the most likely opportunity to detect a significant effect, 450 days, 360 days and 270 days, covering a period that a full trial might realistically cover. A group size of 272 provides power of 0.9 to detect a 10% reduction in the failure rate at significance of 0.05. This is for equally sized groups; Cook and Lawless [62] recommend that when considering failure over time group sizes should be adjusted so that the number of failures is roughly equal across groups and therefore a future trial should inflate the experimental group size by 10%.

Attrition will further influence prospective sample sizes. In this exploratory trial, across the entire project four premises left the study, one withdrew, one closed for refurbishment, one had its license suspended and one permanently closed (due to economic reasons). This attrition occurred towards the end of the follow-up period and did not adversely affect this exploratory trial. While this suggests that it would be perspicuous to assume a 50% rate of attrition for a twelve month follow-up period, refurbishment and temporary suspension of license are temporary and can be accounted for within the methodology described here as a form of censoring. Permanent closure due to a violation of licensable activity is a valid outcome, it is a normal intervention that reduces the incidence of violence to zero, and does not amount to attrition. Furthermore, if the intervention is enforced through existing legislation then voluntary opt-out from routine data collection by police is not optional. Finally, a follow-up period of twelve months is likely the most obvious choice given the power this period affords and that it would further control for any annualised cyclical variation within premises. In sum, a future trial should recruit a minimum of 571 premises and follow them for twelve months post intervention.

##### Breath alcohol concentration

Across both baseline and follow-up surveys a total of 1,999 people provided usable alcometer data, of whom 772 were exiting and leaving a study premises to go elsewhere. Table 1 presents mean BrAC, proportion staggering and surveyors estimated drunkenness by phase and condition for those exiting target premises. One premises requested that customers were not breathalysed and therefore only subjective measures were available for this premises.

**Table 1:** Descriptive statistics

		**Mean (SD) or Proportion**	
		**Baseline**	**Follow-up**
BrAC	Control	53.59 (29.26)	53.68 (31.12)
	Experimental	51.81 (29.80)	54.63 (35.93)
Staggering Gait	Control	0.36	0.33
	Experimental	0.23	0.24
Subjective Drunkenness	Control	5.00 (2.20)	4.89 (2.34)
	Experimental	4.51 (2.20)	4.32 (2.23)
FAST	Control	5.74 (3.53)	5.71 (3.58)
	Experimental	5.83 (3.75)	5.76 (3.52)

A multilevel mixed-effects linear regression (premises nested in location) was specified with phase (baseline and follow-up) crossed with condition (control and experimental). The interaction term yielded an unadjusted coefficient of 3.70 (z = 0.73, p = 0.76). Mean BrAC by phase and condition suggest intoxication did not meaningfully change. A one-way analysis of variance with last premises visited as the categorical independent variable and BrAC as the dependent variable yielded a significant main effect (F(29, 742) = 1.78, *p* < 0.01), an intraclass correlation of 0.03 that in turn yields a design effect of 1.73. These provisional estimates suggest a full trial of RLIs can be expected to detect a significant decrease in levels of intoxication only with a sample size in excess of 11,000. Using the same model, but restricting the BrAC data to baseline and dropping the interaction on phase provides a means of estimating matching across premises. This model yielded no significant effect (z = 0.69, p = 0.75) on BrAC suggesting our null hypothesis that BrAC will be equivalent across control and intervention groups cannot be rejected.

We have argued elsewhere [12,20] that subjective measures of intoxication may provide an alternative to objective BrAC measures; subjective measures are easier to collect and also are more relevant to premises staff who will rely on the physical symptoms of intoxication in order to curtail customers’ excessive intoxication [67]. Three binary indicators of intoxication were collected, whether respondents had glazed eyes, blurred speech or a staggering gait and one index where intoxication was recorded on a ten point Likert scale. Previous work suggests eyes and speech are harder to assess due to noise and low lighting conditions [12,20], means and proportions for the remaining indicators are reported in Table 1. No systematic intervention effect was observed with staggering gait, however a multilevel mixed-effects linear regression (premises nested in location) with phase (baseline and follow-up) crossed with condition (control and experimental) suggested a non-significant reduction in subjective drunkenness, yielding an unadjusted coefficient of −0.32 (z = −0.85, p = 0.80). This change could be attributable to changes in the underlying population, heavy drinking customers may have simply relocated to an alternative premises. The FAST provides one means of controlling for such shifts: 2,014 respondents completed the FAST. A score greater than three (FAST range 0–16) on this screening tool suggests risky drinking patterns. Men typically recorded higher FAST scores (mean = 6.50, SD = 3.56) compared to women (mean = 5.28, SD = 3.32) with 77.74% men and 62.19% women yielding scores indicating that they were risky drinkers. The same multilevel model, controlling for FAST, yielded an adjusted coefficient of −0.15 (z = −0.41, p =0.34). Deriving marginal means yields sample size estimates of 2,048 per group to raise this effect to significance. Although the intraclass correlation coefficient, 0.06, yields a design effect of 2.36 and that the sample size should be raised to 4,833 individuals per group. Sample size estimates on reductions in the hazard rate for violence suggested a total of 272 premises would be required. This translates to a study where approximately a minimum of 17 exiting customers per premises are sampled which is feasible.

The current study assessed whether it was feasible to collect data concerning respondents’ experiences of violence and historical drinking patterns. While the anonymous nature of the survey precluded options to fully validate responses, their inclusion might provide indications of any substantial change in premises’ clientele across premises-level interventions.

##### Experience of violence

2,073 respondents provided responses to the question asking whether they had perpetrated violence in the past twelve months with 21.42% stating that they had; 2, 078 respondents provided responses to the question asking whether they had been a victim of violence in the past twelve months with 43.79% stating that they had. Perpetrating violence was associated with experiencing violence (OR = 6.47, 95% CI 5.07 – 8.26).

#### Sampling biases

Surveyors approached 2,658 people in total, of whom 1,999 provided a usable BrAC score. Previous studies using the current [20] and similar [11] datasets suggest that surveyor estimates of intoxication provide a reasonable indicator of intoxication. It is therefore possible to use surveyor estimates to assess whether intoxication predicted non-response. A *t*-test on estimated intoxication, a 1 (sober) to 10 (severely intoxicated) Likert scale, yielded a significant effect (t = 3.71, *p* < 0.001) with non-respondents estimated as more sober (mean = 4.10, SD = 2.61) than respondents (mean = 4.53, SD = 2.28), replicating an earlier study [12]. We have previously interpreted this observation as one where intoxicated patrons of the NTE have little else to do other than complete surveys having reached satiety.

#### Cost benefit estimates

While this exploratory trial was not designed to provide a full cost benefit analysis of intervention implementation, some provisional estimates can be calculated. The approximate cost of the intervention developed here was £600, a figure based on the auditors involved with this project usual charge for similar work. One average violent incident is estimated to impose costs to health and other services in the UK of £10,407 [68]. Conservatively, one less violent assault would be sufficient to justify expenditure on interventions across 17 premises. If we assume failure (see above) translates to one significant assault (although this likely grossly underestimates the actual rate of serious assault) then assuming a differential in the hazard rate from 0.021 to 0.016 we can estimate the number of premises required to deliver a reduction of one violent incident. For parsimony we will assume a 90 day period which would be expected to yield 1.89 failures in the control group and 1.44 failures in the experimental group, a differential of 0.45 that in turn suggests three intervention premises would be required to demonstrate a reduction in violence of one or more incidents in a three month period and that the intervention is likely to be cost effective. These estimates do not account for a reduction in the harms associated with alcohol misuse.

### Implementation barriers and fidelity

The process evaluation revealed a number of implementation barriers and shortfalls in the dose received, i.e. extent to which premises engaged with the action plans. Premises were reluctant to implement their action plans and to receive follow-up audits. In addition, premises’ perception of risk conflicted with the risk assessments carried out during the preparation phase of the intervention, and their perceptions of the long-term potential of the intervention were low. This occurred despite most premises staff involved with this study stating independently that they had implemented similar actions to those identified within the action plan developed by auditors, although this was disputed by auditors.

Table 2 summarises DPSs reactions to action plans. Of the 107 action points that were included in action plans 14 were implemented. On 17 occasions, premises disagreed with an action point assigned to them because their perception was that the auditor’s assessment was incorrect, or that the item was not a risk factor. On a further nine occasions, premises’ staff disagreed with an action point assigned to them because *informal* procedures were being used where the audit required evidence of *formal* written procedures.

**Table 2:** Intervention DPS reactions to action plans

	**Location 1**	**Location 2**	**Location 3**	**Location 4**	**Total**
Actioned ^1^	2	2	1	1	6
Assessor ^2^	1			1	2
Ambivalent ^3^		2	1		3
Unknown		2^5^	1^4^	2^5^	5

NOTES

^1^ At least one action point implemented, 14 out of 107 action points were implemented in total.

^2^ Premises assessed themselves against the action plan but no action points were implemented.

^3^ Premises read but disregarded the action plan.

^4^ Premises had no recollection of any study activity including receipt of the action plan despite two delivery attempts.

^5^Declined to participate in process evaluation, 29 action points were allocated to these premises.

“if you write everything down there would be paper everywhere, you’d have notices stuck everywhere” [BDPS01]

On 41 occasions, premises agreed that a risk factor was appropriate for the premises but took no action. A recurring theme that was offered as reasons for not implementing action points were subjectivity within the wording, for example, in the assessment of what constitutes ‘intoxication’ with respect to the refusal of service:

“I think we just got to judge the level or what you class as intoxicated: is it someone falling down, is it someone slurring? Is it someone who has just drunk a lot but is ok? Everyone will have different views of what intoxicated is.” [SDPS03]

However, most of the premises reported a range of actions similar to those within the plans that were being (or had been) implemented independently of the study whose objective was to minimise the risk of alcohol related disorder, although the content of actions were not discussed with bar staff.

The intervention incorporated a maintenance phase in which the principle activity was a follow-up audit scheduled for 2–3 months after the first audit. Attempts were made to conduct these audits for the first five matched pairs during which four premises agreed to an on-site audit; a further four agreed to a telephone audit wherein progress against the action plan was discussed; and attempts to contact two further premises failed. Thereafter, intervention auditors took the decision to temporarily suspend follow-up audits. However, during process evaluation interviews, four of the remaining intervention premises reported that they would have agreed to one, either as a learning experience or to co-operate with the study.

“I’d like to see how we stack up to see if anything has changed with implements that we’ve put in” [CDPS03]

“Yeah, they could come in, that’s not a problem” [NDPS02]

Consequently, attempts to conduct follow-up audits were resumed but without success. The auditors acknowledged this process failure while acknowledging that some means of introducing sustainability into the process was essential.

#### Intervention revisions

**Figure 6:**
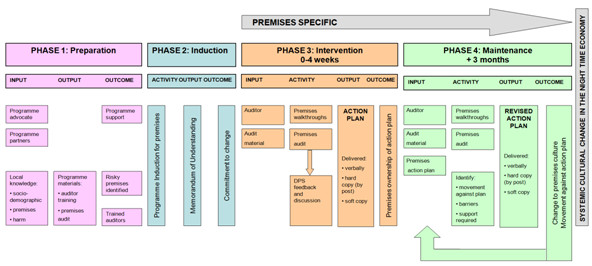
Revised intervention logic model.

Collectively, these issues suggest that the original intervention failed to engage its targets sufficiently. As the affective-motivational elements of an intervention are critical to its success [69], the objective of the revised logic model (see Figure 6) is to address these engagement issues thereby eliminating avoidable threats to intervention fidelity [70]. During the *preparation phase*, the identification of a programme advocate and programme partners was viewed as essential to ensure that the intervention is authoritative and included within (not alongside) local policies [71]. The majority of stakeholders in the NTE and the intervention auditors agreed that the intervention *“…cannot work in isolation of the partners”* (SH02), thus collaborative partnerships would be essential for the governance of the intervention. A local advocate with regulatory authority was also recommended to provide continuity, focus and authority.

None of the premises perceived the audit/walkthrough process to be much of a burden in terms of time or effort. However, despite the fact that those premises included in the study were categorised as high risk premises, most of the DPSs felt that they *“…don’t tend to see too much trouble.”* (SDPS02) and in four cases, participation in the audit was delegated to staff who were neither the DPS, the decision maker nor the interviewee. The intervention auditors reported that this undermined their ability to make an accurate assessment of the premises.

Premises suggested that the opportunity to discuss findings with the auditor before the action plans were produced would be welcomed (n = 7). This view was also expressed by the premises auditors.

The new *induction phase* and post-audit *feedback/discussion* between the auditor and the DPS during the intervention and maintenance phases is therefore intended to facilitate premises’ buy-in to the process. Finally, the duration of the intervention has been extended by allowing iterations of the maintenance phase with the intention of achieving long-term cultural changes within premises thereby reducing their potential to perpetuate existing problems within the night-time economy [72].

## Discussion

The current study evaluated quantitative outcomes for an exploratory RCT of a premises level intervention. It is methodologically feasible to test a RCT of a premises-level intervention and data are available to test outcomes. Street surveys remain one of the few feasible means of collecting individual level intoxication data. While alcometers provide objective data, it may be more cost- effective to use observational data from surveyors to assess levels of intoxication, a method that has ecological validity as presumably serving staff use similar physiological manifestations, such as a staggering gait, to infer customers’ levels of intoxication. A sufficient number of responses were collected on a per-premises basis to enable the formal analysis of survey data. Further, it was possible to collect reliable data on secondary measures, such as FAST and respondents’ experiences of violence, to better understand how the intervention might contribute to dispersal. Similarly, data from surveyors were sufficient to determine response biases to the survey, in particular whether non-response was associated with respondent intoxication. While the NTE presents a difficult environment in which to conduct experimental work, descriptive statistics from our street surveys strongly suggest that this method offers a potential method to test intervention effectiveness. This is mitigated, however, by a large sample size requirement for BrAC measures and associated costs involved with placing researchers around premises over the projected twelve month follow-up period. This study required a team of six collecting data across four sessions to monitor each matched pair of premises. Each session lasted six hours incurring a cost of 60 staff-hours per premises. There are additional costs in recruiting, screening and administrating the staff required to undertake such work.

Analysis of police data suggest a full implementation trial might be expected to yield a reduction in the rate of premises-level violence. Failure was defined as a period in which premises were open and one, or more, violent incident was recorded. This analytic strategy was adopted because police data do not readily indicate the relationship between events and individuals in such a way that they can be disambiguated. Our parsimonious approach was to simply assume that premises level risks can lead to one or more incidents and that therefore one or more violent events indicate premises were in a state of failure across that session. These assumptions, together with additional questions on whether intervention effectiveness might wane over time placed requirements on available analytic strategies such that a derivation of the Cox model was most appropriate. These matters are discussed more fully elsewhere [18]. In sum, police data can be used to assess violence at the premises-level, appropriate analytic strategies are available.

Our analysis of police data suggested that the intervention led to non-significant hazard ratio of 0.9. Further power analyses indicate a twelve month follow-up period across 517 premises would be required to detect a significant improvement and that such an intervention is likely to be cost efficient. In the UK at least, it is very unlikely that one city will contain 517 at-risk premises. This Project was based in Wales and police conservatively estimate that there are at least 1,500 risky premises in Wales that are accountable for approximately 14,000 assaults per annum. A future trial would therefore be realistic across a larger area than one city location. While the RLI described here may not have a considerable impact locally, the robust methodology and well defined outcome measures means that the adoption of procedures described here provides verification of cost savings aggregated across larger areas.

There was minimal input from statutory authorities, such as the police and Local Authorities, in the delivery of the intervention. A recent systematic review of premises-level interventions found that enforcement can play an important role in intervention delivery. This is highlighted by the Traffic Light Scheme [24].

Data from this police led scheme, while not having been subjected to rigorous evaluation, suggested a 70% reduction in violence for target premises. In the UK the police have powers over premises that include the imposition of conditions as part of the premises’ license. Therefore, our observed hazard ratio might be considered to be at the lower end of the intervention effectiveness spectrum and one where the dose would increase if the intervention was enforced.

Our analysis of alcometer data did not suggest any consistent intervention effect although a modest reduction in one subjective measure of intoxication was found. This study was the first to use objective measures of intoxication recorded from study premises customers as an outcome measure and due to the current lack of data pertaining to factors that promote alcohol misuse in the NTE it is unclear whether addressing premises-level risks should be expected to affect a noticeable change in drinking behaviour. Undoubtedly, consumption will be motivated by the confluence of individual predispositions and idiosyncrasies, their perception of the normative environment, social and peer pressure, availability and premises characteristics including promotions. Indeed, our recent analysis of premises characteristics that might promote violence found that premises whose customers showed a greater increase in intoxication and that premises with alcohol promotions were those most likely to have recorded a higher level of violence [20]. These observations provide evidence for a linkage between serving practices and alcohol-related violence, and that therefore premises-level interventions may be effective in reducing both alcohol misuse and violence. On balance, the data presented here does suggest that premises should continue to be considered as a point of intervention for both violence and alcohol misuse, particularly as FAST data indicate that more than 60% of drinkers are drinking at risky levels.

A critical question emerging from the process evaluation is whether the non-significant reduction in violence, from which effect sizes were calculated, is realistic and can be used to inform future trials. As a portion of actions described in the intervention were implemented and DPSs stated that adequate processes were in place in some cases then it is conceivable that the intervention worked as a form of “nudge” [73], reinforcing obligations of which premises staff were already aware.

However, auditors disagreed that risk reduction strategies were already in place in many premises and in others there was some reluctance on the part of premises staff to, first, admit failings in their premises and, second, for senior staff to take responsibility for change. Some refinement of the intervention is therefore necessary as a first stage of any definitive trial to identify an appropriate regulatory partner that could act as an advocate and provide ongoing support. A series of approaches to promote ongoing DPS engagement with the intervention have also been identified including an induction phase, strategies to promote feedback and discussion between auditors and premises and an extended period of maintenance activities.

The intervention effect is partially consistent with the intended effect on violence. For alcohol misuse, however, the direction is not consistent. We suggested that validated subjective indices of intoxication provide realistic outcome measures as it is these that servers and premises staff are most likely to rely on in respect of proactive serving practices. To this extent one subjective measure yielded a change in the direction that was expected but another, staggering gait, did not. This is in turn mediated by what might be regarded as differences between control and intervention premises in respect of their customers’ baseline levels of intoxication. While premises were closely matched by violence rates across the twelve months preceding study implementation there was some discrepancy in the proportion of those customers staggering at baseline, suggesting that while serving practices and customer intoxication appear associated with premises violence rates [20] and subjective measures of intoxication are good proxies of objective measures of intoxication [12], the reasonably short period across which baseline measures were taken (one weekend for each premises) might deprecate the stability of intoxication measures. It is probably not cost effective to implement a longer data collection period for alcometer data (see above). While street survey data are valuable in providing a better understanding of alcohol misuse in the NTE [11,22], the costs for a large-scale roll-out would be substantial.

In sum, it is methodologically feasible to implement premises-level trials to test interventions in the night time economy, secondary data such as police or hospital-recorded assaults provide reliable outcomes measures. Intervention dose would be improved if delivered through a statutory partner, as designated premises supervisors are mostly uninterested in harm reduction strategies. Success would be further enhanced if staff across the entire socio-ecology of premises were engaged.

## Competing interests

The authors declare that they have no competing interests.

## Authors' contributions

SCM, SM and LM conceived of the study. SCM, SM, LM and JPS contributed to the original proposal from which the protocol was developed. SCM, SM, LM, EB, SNM and IRB were responsible for the conduct of the trial with SCM as principal investigator. IRB was responsible for the day to day management of the trial. EB and SNM took responsibility for and SM managed the process evaluation. SCM, IRB, SM, EB, LM and SM drafted the manuscript. All authors read and approved the final manuscript.

## Pre-publication history

The pre-publication history for this paper can be accessed here:

http://www.biomedcentral.com/1471-2458/12/412/prepub

## Supplementary Material

Additional file 1 **Appendix 1. **Street survey. Click here for file
