# Supplementary figures and images for: An exploratory randomised controlled trial of a premises-level intervention to reduce alcohol-related harm including violence in the United Kingdom
Source: BMC Public Health. 2012 Jun 7;12:412. doi: 10.1186/1471-2458-12-412 (PMC3537579; doi:10.1186/1471-2458-12-412)

Appendix 1 - Street Survey

The Street Survey used to collect some of the data presented here


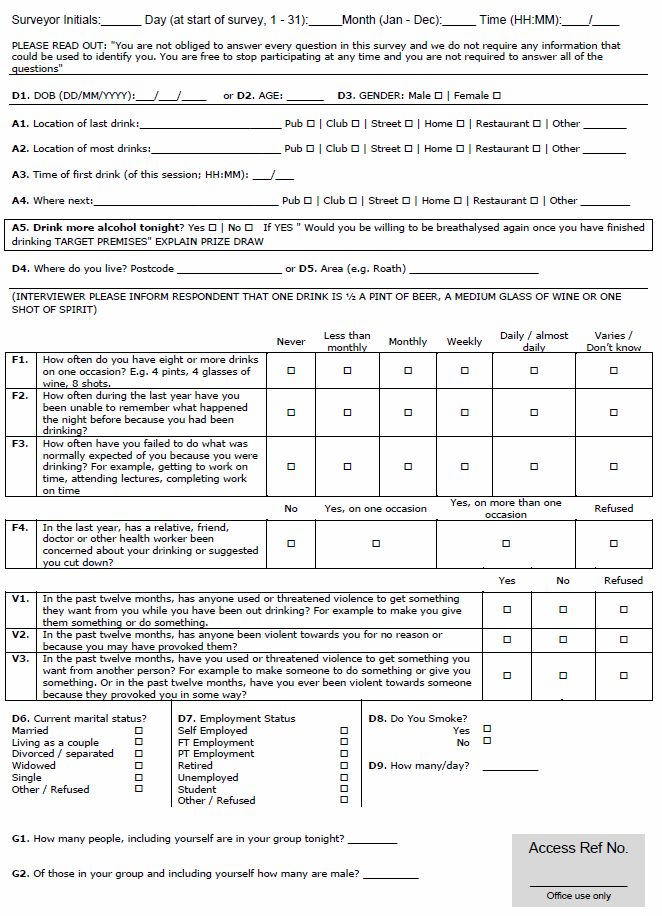


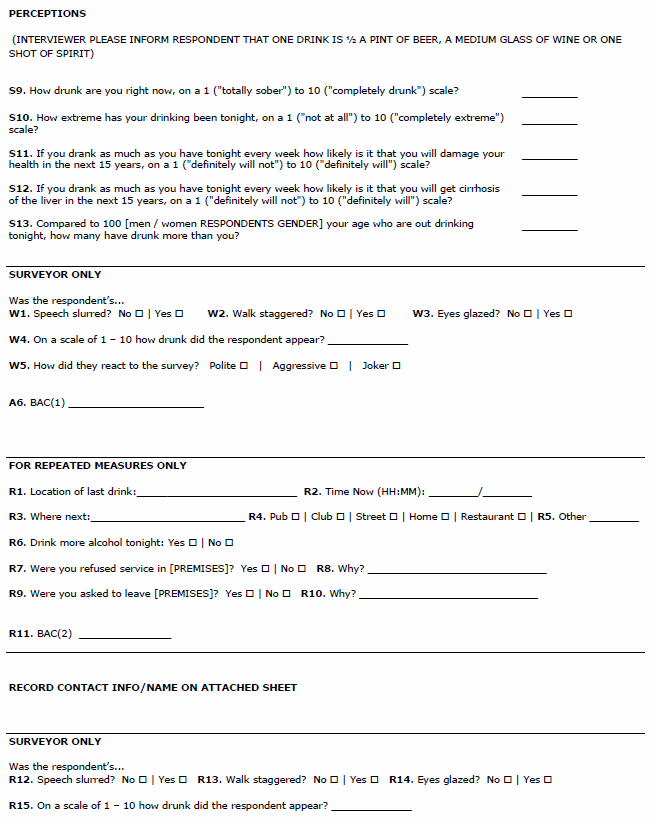

Supplement: Additional file 1 — Appendix 1. Street survey. [file 1471-2458-12-412-S1.doc]
